# Metabolomics identifies a biological response to chronic low-dose natural uranium contamination in urine samples

**DOI:** 10.1007/s11306-013-0544-7

**Published:** 2013-05-21

**Authors:** Stéphane Grison, Gaëlle Favé, Matthieu Maillot, Line Manens, Olivia Delissen, Eric Blanchardon, Nathalie Banzet, Catherine Defoort, Romain Bott, Isabelle Dublineau, Jocelyne Aigueperse, Patrick Gourmelon, Jean-Charles Martin, Maâmar Souidi

**Affiliations:** 1 Institut de Radioprotection et de Sûreté Nucléaire (IRSN), PRP-HOM, SRBE, LRTOX, Fontanay-aux-Roses, France; 2 Institut de Radioprotection et de Sûreté Nucléaire (IRSN), PRP-HOM, SDI, LEDI, Fontanay-aux-Roses, France; 3 Institut de Radioprotection et de Sûreté Nucléaire (IRSN), PRP-HOM, Fontanay-aux-Roses, France; 4 Aix Marseille Université, NORT, 13005 Marseille, France; 5 Inserm, UMR_S 1062, 13005 Marseille, France; 6 Inra, UMR_INRA 1260, 13005 Marseille, France

**Keywords:** Uranium, Low-dose, Chronic, Metabolomics, Urine, *N*-methylnicotinamide

## Abstract

Because uranium is a natural element present in the earth’s crust, the population may be chronically exposed to low doses of it through drinking water. Additionally, the military and civil uses of uranium can also lead to environmental dispersion that can result in high or low doses of acute or chronic exposure. Recent experimental data suggest this might lead to relatively innocuous biological reactions. The aim of this study was to assess the biological changes in rats caused by ingestion of natural uranium in drinking water with a mean daily intake of 2.7 mg/kg for 9 months and to identify potential biomarkers related to such a contamination. Subsequently, we observed no pathology and standard clinical tests were unable to distinguish between treated and untreated animals. Conversely, LC–MS metabolomics identified urine as an appropriate biofluid for discriminating the experimental groups. Of the 1,376 features detected in urine, the most discriminant were metabolites involved in tryptophan, nicotinate, and nicotinamide metabolic pathways. In particular, *N*-methylnicotinamide, which was found at a level seven times higher in untreated than in contaminated rats, had the greatest discriminating power. These novel results establish a proof of principle for using metabolomics to address chronic low-dose uranium contamination. They open interesting perspectives for understanding the underlying biological mechanisms and designing a diagnostic test of exposure.

**Electronic supplementary material:**

The online version of this article (doi:10.1007/s11306-013-0544-7) contains supplementary material, which is available to authorized users.

## Introduction

Above and beyond acute accidental pollution by various substances, the chronic low-rate dispersion of pollutants to the environment is a serious health issue that raises questions about the effects on humans of such continuous low-grade exposure. The potential health repercussions are not always satisfactorily addressed. Thus both the public and more directly involved stakeholders request studies to evaluate the environmental risks related to exposure to these various low-grade pollutants (European-Commission 2011). Radionuclides are among the most interesting of the environmental chemical compounds for experimental studies because of the societal concerns they raise. They can be dispersed after major nuclear power plant accidents (for example, cesium 137) (Gagnaire et al. 2010) or by military activity with depleted uranium (Handley-Sidhu et al. 2010). Other radionuclides are naturally occurring elements present in rocks, water, and the food chain, such as uranium (Bleise et al. 2003; Craft et al. 2004), which can also be dispersed in higher quantities by pit mining (Arogunjo et al. 2009). The consequences of acute high doses of uranium on health are relatively well known (Kathren and Burklin 2008) and have been described in particular in kidneys. However, the long-term effects of low-dose uranium contamination remain unknown and require more investigation. Several in vivo studies have shown that uranium can affect various physiological systems, including intestinal inflammatory pathways, the reproductive system, brain pro- and anti-oxidant activity, and xenobiotic metabolisms (Briner 2010; Dublineau et al. 2006, 2007; Feugier et al. 2008; Lestaevel et al. 2009; Paquet et al. 2006; Souidi et al. 2009; Wade-Gueye et al. 2012). Nonetheless, a still more sensitive approach is necessary to overcome the specific limitations due to low doses. Metabolomics, a noninvasive technology suitable for clinical studies (Zhang et al. 2012; Corona et al. 2012; Senn et al. 2012), has recently been proven quite powerful for identifying a discriminant metabolic signature of chronic low-dose cesium 137 contamination in rats (Grison et al. 2012). The aim of this study was thus to use a metabolomic approach, combined with conventional toxicology, to characterize the biological impact of chronic low-dose exposure to natural uranium (_nat._U) in rats by an exhaustive metabolite analysis and to attempt to decipher potential underlying molecular mechanisms. To mimic environmental contamination of drinking water, especially among children, who are known to be a sensitive subgroup in toxicology (Schwenk et al. 2003; Wang and Pinkerton 2007) and radiobiology (De Santis et al. 2007; Magdo et al. 2007), rats were exposed from birth to adulthood through drinking water containing a concentration of _nat._U known not to be toxic to the kidneys. Standard clinical and biological characteristics, including body and organ weight, food and water consumption, blood cell counts, and plasma and urine biochemistry were assessed to verify the absence of toxicity. At the same time, we used liquid chromatography–mass spectrometry (LC–MS) to acquire exhaustive urine metabolite profiles, which we analyzed by multivariate statistical methods.

## Materials and methods

### Animals and contamination procedure

#### Animals

Sprague–Dawley rats, 12 weeks old and 16 days pregnant, were obtained from Charles River Laboratories (L’Arbresle, France). They were housed individually and maintained in a 12-h light/12-h dark cycle (regular cycle) at 21 °C and 50 % humidity, with access to a standard rodent pellet diet and water ad libitum. After weaning, male offspring were housed, each paired with a rat from a different mother (assigned by randomization). Female offspring and mothers were euthanized. All experimental procedures were approved by the Animal Care Committee of the Institute of Radioprotection and Nuclear Safety (IRSN) and complied with French regulations for animal experimentation (Ministry of Agriculture Act No. 87-848, October 19, 1987, modified May 20, 2001).

#### Contamination procedure

Rats were contaminated through their drinking water: _nat._U in a solution of uranyl nitrate hexahydrate (UO_2_(NO_3_)_2_·6H_2_O) was prepared to a final uranium concentration of 40 mg L^−1^ (daily uranium intake: 1 mg/rat/day) and dissolved in mineral water. This dose corresponds to three time as much as the highest uranium concentration found naturally in well waters in Finland (Salonen 1994). _nat._U (Olympic) was obtained from CERCA (Pierrelatte, France). Control animals drank uncontaminated mineral water. The specific activity of the _nat._U was 2.42 × 10^+4^ Bq/g and its isotopic composition was ^238^U = 99.307, ^235^U = 0.688, and ^234^U = 0.005 %. Mothers of the offspring in the treated group were also exposed to _nat._U through drinking water from birth to euthanasia (mothers of the control rats received uncontaminated mineral water). Until weaning, offspring were theoretically contaminated by mother’s milk [human offspring receive ~5 % of the mother’s daily dose (Wappelhorst et al. 2002)]. The food and water intake of both groups were monitored weekly throughout the contamination period.

### Collection of organs and biofluids

One morning when the rats were 9 months old, they were placed in metabolic cages (one per cage), with access to a rodent ground pellet diet and water (contaminated or not) ad libitum. Urine was collected twice a day for 48 h, with sodium azide (0.01 %) added to prevent bacterial growth (Griffin et al. 2001), and refrigerated at +4 °C. Each rat’s fractions were pooled, mixed, and centrifuged; supernatants were frozen at −80 °C. The rats were then moved to conventional cages (one per cage) with food and drink ad libitum until the evening to reduce stress. To control the diet cycle, food was removed in the evening until the next morning. Five hours before euthanasia, around 12 g of standard rodent pellet food was added directly to each cage to stimulate the desire to eat before sleep. Rats were anesthetized by inhalation of 5 % isoflurane (Abbot France, Rungis, France) and euthanized by intracardiac puncture, with blood collected in heparinized tubes. Whole blood was centrifuged (5,000 rpm) and plasma supernatants were immediately frozen at −80 °C. Organs were dissected on ice, weighed, deep-frozen in liquid nitrogen, and stored at −80 °C.

### Biological and uranium analyses

#### Measurement of biological characteristics

##### Blood cell counts

Complete blood counts (CBC) were performed with an MS-9 vet automatic counter (Melet-Schlossing, Osny, France). Remaining blood was centrifuged at 400 g for 10 min, and plasma was frozen (−80 °C) for later use.

##### Biochemical panel

Biochemical measurements were made from thawed plasma samples by an automated spectrometric system (Konelab 20 from Thermo Electron Corporation, Cergy-Pontoise, France), with the manufacturer’s biological chemistry reagents and protocols. The markers measured in plasma included glucose, albumin, total protein, lactate dehydrogenase (LDH), alkaline phosphatase (ALP), cholesterol, high-density lipoprotein (HDL), low-density lipoprotein (LDL), phospholipids, triglycerides, alanine aminotransferase (ALT), aspartate aminotransferase (AST), total and direct bilirubin, transferrin, ferritin, ceruloplasmin iron, phosphorus, calcium, chlorine, potassium, magnesium, sodium, urea, creatinine, and creatine kinase.

#### Measurement of uranium accumulation and dose calculation

##### Measurement of natural uranium organ contamination

Samples were mineralized (Ejnik et al. 2000) and then analyzed for their uranium content by ICP-MS (XSERIE 2, Thermoelectron, France). A multielement standard solution (Analab, France) was used to optimize experimental conditions and apparatus parameters to obtain the best signal/noise ratio for ^238^U. In all solutions likely to be analyzed (biological samples or calibration solutions), bismuth 209 was added as an internal standard at 1 μg L^−1^. Six standard solutions for the calibration curve (0, 0.005, 0.01, 0.1, 0.5, and 1 μg L^−1^) were freshly prepared by dilution of a standard solution at 10 mg L^−1^ in 2 % nitric acid (NORMATOM for trace metal analysis, VWR Prolabo). A linear relation—count number (^*i*^U) = *f*([^*i*^U])—can thus be set up for each isotope, *i* = [235; 238] with [^*i*^U] equal to the isotope concentration in μg L^−1^. Isotopy and dosage reliability were regularly verified with standard solutions (six quality controls of different concentrations and isotopy distributed throughout the analysis). Blank samples were run every five samples to check the stability of the background and prevent potential contamination. For ^238^U, the detection and quantification limits were respectively 0.5 and 1.5 ng/L, and for ^235^U, 0.01 and 0.03 ng/L. The limits for ^238^U were applied to total uranium.

##### Renal dose estimation (9 months post-natal)

The radiation yields and energies of radiation emitted in nuclear transformation of the isotopes forming _nat._U were taken from ICRP (1983). Alpha particles and Auger and internal conversion electrons were assumed to be locally absorbed in the source organ. Photons were transported with the code MCNPX in a voxel phantom of an adult male rat from the same strain. In view of the preponderant concentration of _nat._U in kidneys (Table 1) and the small fraction of energy emitted as penetrating radiation, kidney irradiation by _nat._U from the rest of the body was ignored because negligible. The absorbed dose rate to the kidney was thus determined at 9 months of age based on the kidney concentration of _nat._U and kidney mass indicated in Table 1 and assuming a homogeneous concentration of _nat._U throughout the entire 9 months.

**Table 1:** Mean ± SEM of (i) uranium concentration in plasma, urine and kidneys, (ii) dietary consumption measured over the 4 months before euthanasia, (iii) whole-body weight, kidney weight and mean ratio of kidney to whole-body weight in each group, (iv) 48-h urine collection, (v) plasma enzymes, proteins, carbohydrates, lipids, ions and other metabolites, (vi) urine proteins, carbohydrates, ions, other metabolites and creatinine clearance evaluation in control and contaminated (_nat._U) groups after 9 months of chronic radionuclide ingestion through drinking water (40 mg/L)

Uranium concentrations	Control (10)	_nat._U (10)	Plasma analysis	Control (9)	_nat._U (10)
Plasma (ng U/g)	0.86 ± 0.05	0.92 ± 0.06	ALAT/GPT (U/L)	37.2 ± 4.5	36.5 ± 2.6
Kidney (ng U/g)	5.45 ± 0.64	358.94 ± 84.02***	ASAT/GOT (U/L)	69.8 ± 4.6	89.5 ± 5.0**
Urine (ng U/g)	2.00 ± 0.24	14.99 ± 1.08***	CK (U/L)	184.0 ± 19.9	259.9 ± 36.2
Dietary consumption			CK-MB (U/L)	335.2 ± 44.9	386.2 ± 42.5
Drinking (mL/day/rat)	27.1 ± 0.3	28.8 ± 0.2	LDH (U/L)	512.2 ± 87.1	532.5 ± 56.2
Food consumption (g/day/rat)	28.0 ± 0.3	26.0 ± 0.3	ALP (U/L)	76.7 ± 4.7	66.4 ± 5.4
Weight measured at sacrifice			CERU (mg/L)	98.3 ± 7.7	91.6 ± 10.0
Total body (g)	615 ± 26	563 ± 18*	CHE (U/L)	154.6 ± 43.1	98.2 ± 6.9
Kidney (g)	1.72 ± 0.04	1.77 ± 0.05	Albumin (g/L)	30.4 ± 0.4	30.7 ± 0.6
Ratio kidney/total body (%)	0.28	0.32*	Total proteins (g/L)	61.4 ± 1.0	54.7 ± 5.9
48-h urine collection			Transferrin (g/L)	1.5 ± 0.1	1.5 ± 0.0
Urine volume (g)	28 ± 2	32 ± 1	Glucose (g/L)	11.7 ± 0.6	11.8 ± 0.4
			Triglycerides (mM)	1.0 ± 0.2	0.9 ± 0.1
Urine analysis	Control (10)	_nat._U (10)	Cholesterol (mM)	2.5 ± 0.2	2.4 ± 0.2
Amylase (U)	335.90 ± 24.60	342.47 ± 21.12	HDL-cholesterol (mM)	1.1 ± 0.1	1.2 ± 0.1
Glucose (mmol)	0.06 ± 0.01	0.06 ± 0.00	LDL-cholesterol (mM)	0.4 ± 0.0	0.3 ± 0.0
Calcium (mmol)	0.06 ± 0.00	0.08 ± 0.01	Phospholipids B (g/L)	1.5 ± 0.1	1.4 ± 0.1
Potassium (mmol)	0.55 ± 0.28	1.56 ± 0.35**	Calcium (mM)	2.6 ± 0.1	2.6 ± 0.0
Sodium (mmol)	0.63 ± 0.33	1.71 ± 0.39**	Iron (µM)	21.8 ± 1.3	23.4 ± 1.4
Phosphorus (mmol)	0.81 ± 0.10	0.55 ± 0.07	Chlorine (mM)	93.0 ± 1.9	93.8 ± 1.0
Total proteins (g)	0.11 ± 0.03	0.08 ± 0.02	Potassium (mM	4.2 ± 0.1	4.5 ± 0.2
Urea (mmol)	26.56 ± 2.08	26.23 ± 1.11	Magnesium (mM)	0.7 ± 0.0	0.6 ± 0.1
Uric acid (µmol)	45.97 ± 2.22	47.51 ± 2.80	Phosphorus (mM)	1.3 ± 0.1	1.4 ± 0.1
Creatinine (µmol)	333.17 ± 19.83	328.79 ± 20.41	Creatinine (µM)	43.7 ± 1.0	47.6 ± 1.5*
Creatinine clearance (ml/min/kg)	4.4 ± 0.2	4.3 ± 0.2	Direct bilirubin (µM)	3.0 ± 0.3	3.0 ± 0.2
			Total bilirubin (µM)	2.1 ± 0.3	2.3 ± 0.2
			Urea (mM)	5.7 ± 0.2	5.7 ± 0.2

The number of rats for each measurement is indicated in parentheses

Results are significantly different for: * *P* < 0.05; ** *P* < 0.01; *** *P* < 0.001

#### Statistical analysis

This animal protocol of _nat._U contamination used two groups each of 10 rats. Health diagnostic values were reported as means ± standard errors of the means (SEM). The control and contaminated groups were compared with student’s *t* test in normal populations or the rank sum test in non-normal populations. Statistical significance was defined by a *P* value < 0.05. Statistical analyses were performed with Sigmastat statistical software (SPSS, Paris, France).

### Metabolomic analysis

#### Sample preparation

Urine samples were diluted immediately before analysis. After centrifugation for 15 min at 11,000 rpm and 4 °C, 100 μL of thawed urine was mixed and shaken for 1 min with 400 μL of deionized water. After a second centrifugation for 5 min at 3,000 rpm, 50 μL of supernatant was transferred into HPLC vials for analysis.

To check for data quality, a blank sample (deionized water) and a pool sample (a mixture of all samples) were extracted/diluted and analyzed repeatedly along with the sample series.

#### LC–MS analyses

The samples were analyzed on an Agilent 1200 RRLC coupled to a Bruker microTOF ESI-hybrid quadrupole-time of flight mass spectrometer (Wissembourg, France).

For polar compounds from plasma, the LC conditions were: autosampler temperature, 4 °C; column type, EC 100/2 Nucleodur C18 pyramid; particle size, 1.8 μm (Macherey–Nagel, Les Ulis, France); column temperature, 40 °C; solvent flow, 0.4 mL/min (solvent A: 95 % water, 5 % acetonitrile, 0.1 % formic acid, and solvent B: 95 % acetonitrile, 5 % water, 0.1 % formic acid); gradient, 3 % B for 1 min, 3–50 % B for 2 min, 50–100 % B for 6 min, 100 % B for 2 min, 100–3 % B for 1 min, and 3 % B for 4 min (running time, 16 min).

The MS conditions were as follows: acquisition mode, positive electrospray ionization (ESI+) and full scan 50–1,500 *m*/*z*; capillary voltage, 4.5 kV; capillary temperature, 200 °C; cone voltage, 40 V; drying gas flow, 9.5 L min^−1^; nebulizing gas pressure (nitrogen), 2.9 bar.

For metabolite identification purpose, MS/MS analyses were performed on selected urine samples and standard molecules. LC and MS methods were used for the MS analyses, and MRM parameters were set at 8.0 for isolation width, 2.0 for acquisition factor, and 10.0, 20.0 and 30.0 eV for collision energies. Creatine (MET250A), 4,6-dihydroxyquinoline (424048—250MG), and 5-hydroxyindoleacetate (H8876—100MG) were purchased from Sigma (St. Quentin Fallavier, France) and N1-methylnicotinamide (M0375—10 g) from TCI Europe (Zwijndrecht, Belgium). Solutions of 1 mg/mL were prepared in different solvent mixtures according to each molecule’s Log *P* value (water with 0.1 % formic acid for creatine, with 2 % acetonitrile for N1-methylnicotinamide, and with 10 % acetonitrile for the others) and injected at a 10 μg/mL concentration.

#### Data preprocessing and filtering

LC–MS raw data were exported to “.cdf” file format with the manufacturer’s software DataAnalysis (Bruker, Wissembourg, France) and preprocessed with the freely available XCMS software including the CAMERA script (Smith et al. 2006). Peak picking was performed with the ‘centWave’ method (‘peakwidth’ parameter reduced to 3–15 s to fit to UPLC performance, and ‘snthresh’ to 5 to detect more peaks), retention time correction with the obiwarp method (‘profStep’ reduced to 0.1 *m*/*z* as recommended for QTOF mass spectrometers), peak grouping with ‘bw’ and ‘mzwidth’ parameters reduced to 5 and 0.025, respectively, and gap filling with the default parameters.

To check for and ensure data quality, three successive filtering steps were applied to the pre-processed data using an in-house script ran on R, based on the signal/noise ratio of each MS feature in the pool samples (cutoff set at 5 between features matching both in biological and blank samples), the coefficient of variation of the intensity of the variables in the pool samples (cutoff set at 20 %) and the coefficient of the autocorrelation of the variables in a given ‘pcgroup’ (cutoff set at 0.8). Preprocessing and filtering of the raw LC–MS data reduced the number of variables from 3,446 to 1,376 (40 %) in urine.

#### Statistical analyses

The most discriminatory metabolites were sought in the dataset based on partial least-square discriminant analysis (PLS-DA) using the variable importance in projection (VIP) procedure. All multivariate statistical analyses were performed with SIMCA-P+ 12.0 software (Umetrics, Umeå, Sweden).

For each discriminatory compound, a receiver operating characteristic (ROC) curve as well as the area under this ROC curve (AUC) were computed with the R package pROC. A 95 % confidence interval was assessed for sensitivity with 2000 bootstrap replicates. A boxplot by group was calculated for each compound selected.

#### Metabolite identification

The 40 most discriminatory metabolites were searched in the MZedDB, a web browser screening several databases simultaneously, for tentative annotation using the accurate measured mass (accuracy <15 ppm) (http://maltese.dbs.aber.ac.uk:8888/hrmet/index.html) (Draper et al. 2009) and the Brüker Data Analysis molecular formula engine, using isotopic patterns. The KEGG compound ID of any hits was recorded, and all recorded IDs were inserted into the KEGG Mapper (http://www.genome.jp/kegg/tool/map_pathway2.html) for tentative pathway identification. For the compounds involved in the first four proposed pathways, a standard molecule was purchased, when available, and subjected to MS/MS analysis. We compared spectral data obtained with standard molecules and experimental samples to one another and searched for matches in the Metlin (http://metlin.scripps.edu/spec_search.php), MassBank databases (http://www.massbank.eu/MassBank/), or HMDB (http://www.hmdb.ca/spectra/ms/search).

## Results

### Uranium intake and dose calculations

Results confirmed that the kidney is a preferential target organ for uranium accumulation (Table 1); uranium concentration was very low in plasma (close to the natural background) and significantly higher in urine.

At 9 months of age, the absorbed dose rate in the kidneys of the contaminated rats was estimated at 5.4 × 10^−7 ^Gy d^−1^. Under the maximizing assumption of a constant dose rate over 9 months, the maximum dose absorbed by the kidneys at sacrifice should still be as low as 0.15 mGy (considering a homogeneous concentration of _nat._U throughout the entire 9-month period).

### Effect of uranium contamination on biological characteristics

We monitored drinking water and food consumption during the 4 months preceding the end of the contamination (once a week). Control and contaminated rats did not differ in their food consumption or water intake (Table 1).

To identify any toxic morphological changes, we weighed the whole body and one kidney (on the same side for each) for all rats and compared the groups. Calculation of relative organ weights showed a slight statistical difference but excluded any appreciable physiological effect (Table 1).

No difference between the groups was observed for the urine volume collected during the 48-h period shortly before sacrifice (Table 1).

Blood counts did not differ between the groups after contamination (supplemental Table 1).

Several biochemical markers in plasma were also assessed, including proteins, ions, and liver, cardiovascular, and kidney markers (Table 1). Except for slightly elevated AST-GOT (*P* ≤ 0.01) and creatinine (*P* ≤ 0.05) levels in the contaminated rats (nonetheless within the physiological range), no differences were observed between the groups.

Similarly, urine sodium and potassium levels were statistically higher in contaminated than in control rats, but remained in the normal range as did the measurement of creatinine clearance, which showed no significant difference between the groups (Table 1).

### Effect of uranium contamination on urinary metabolites

#### Discrimination between control and contaminated rats by urine samples

**Fig. 1:**
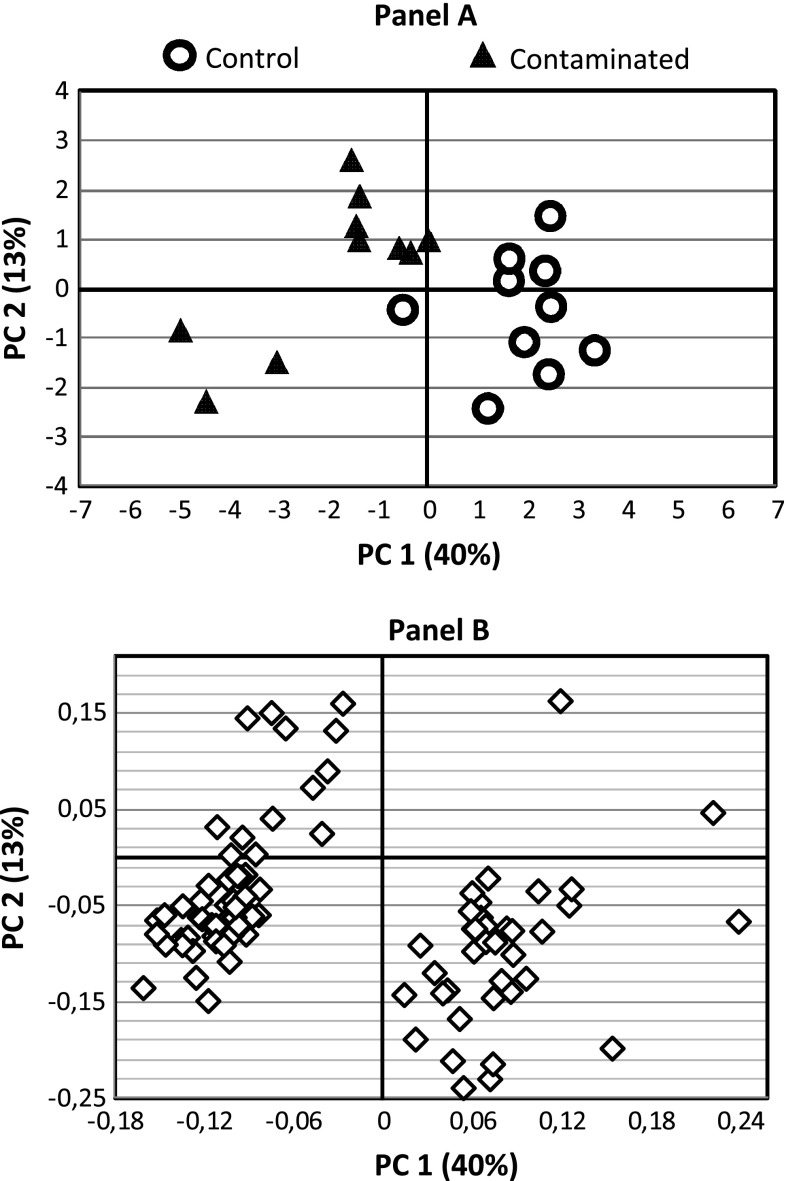
Principal component analysis (PCA) performed on the 95 most discriminatory urine metabolites (VIP score ≥ 1.8 in the PLS-DA model built with the 1,376 urine metabolites). **a** Score scatter plot; PC1 and PC2 describe 40 and 13 % of the total variance, respectively. **b** Loading scatter plot; 60 % of the discriminant features characterize the contaminated rats (*left-hand side* on the plot)

**Fig. 2:**
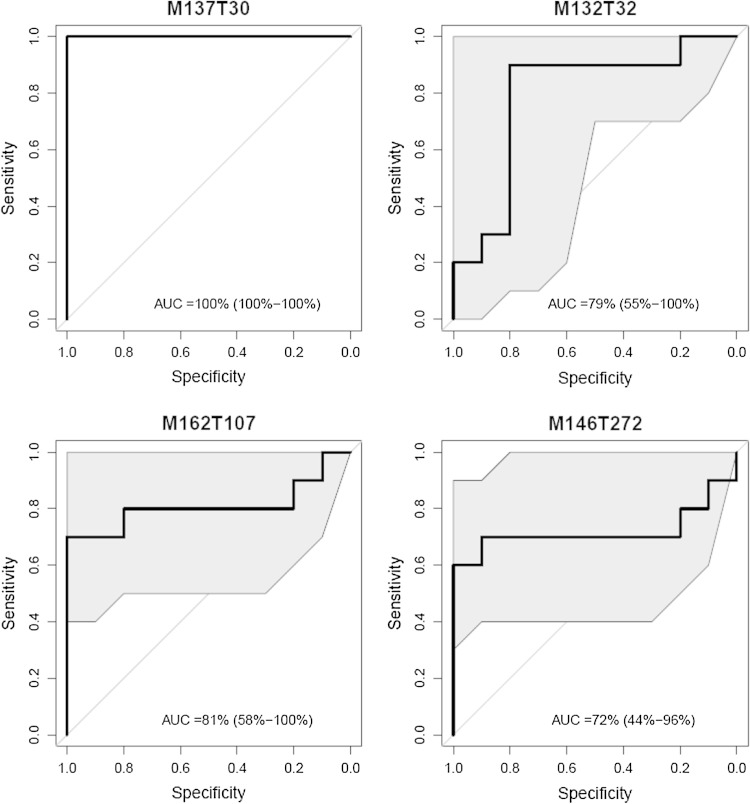
Receiver operating characteristic (ROC) curve and area under the curve (AUC) of the 4 metabolites identified among the top 40 for discriminating the control from the contaminated rats. According to their VIP score, these metabolites were ranked 1st (M137T30), 6th (M132T32), 11th (M162T107) and 32th (M146T272)

The PLS-DA performed on the 1,376 features detected in urine samples resulted in robust, validated discrimination between the control rats and those contaminated with the _nat._U (R2 = 92 %, Q2 = 55 %, CV-ANOVA = 0.009). The 95 most discriminating variables, selected according to their VIP score (above 1.8), were used for another PCA. The first principal component described 40 % of total variance and could be interpreted as the inter-group variation, while the second principal component described 14 % of total variance and showed the interindividual variability (Fig. 1a). Of the discriminant features, 60 % were characteristic of the contaminated group (Fig. 1b). Using the same 95 features, a new PLS-DA model was fitted to identify the top 40 discriminating features (Table 2). According to the PCA results, only one PLS-DA component was necessary for significant discrimination between control and contaminated rats (R2 = 89 %, Q2 = 74 %, CV-ANOVA <0.0005) (plot not shown). The discriminatory power of the top 40 features is quite satisfactory as their AUC under the ROC curve was above 71 % (above 80 % for the top 27). The AUC of the first discriminatory metabolite was even 100 % (Fig. 2).

**Table 2:** Top 40 metabolites involved in discriminating between the control and contaminated rats

LC–MS ID	Sample status^a^	Generated formula^b^	Adduct	Sigmafit^c^	ppm	Chemical formula	Identification	Database ID	Function
M137T30	Control	C_7_H_9_N_2_O	[Ml+.]1+	10.5	4.7	C_7_H_8_N_2_O	1-Methylnicotinamide	C02918	Nicotinate and nicotinamide metabolism, regulate thrombotic as well as inflammatory processes in the cardiovascular system
M340T269	Control	C_9_H_10_NO_2_	[M+H]l+	0.7	5.8	C_9_H_9_NO_2_	4-(3-Pyridyl)-3-butenoic acid moiety of glycine-cysteine conjugate	HMDB01424	Nicotine degradation II and nicotine degradation III
M136T46	_nat._U	C_7_H_6_NO_2_	[M−NH_3_+H]1+	5.6	7	C_7_H_8_N_2_O_2_	Nl-Methyl-2-pyridone-5-carboxamide	HMDB04193	Nicotinate and nicotinamide metabolism end products of nicotinamide-adenine dinucleotide (NAD) degradation
M338T217	Control	C_9_H_8_NO_2_	[M+H]l+	3.9	11.4	C_9_H_7_NO_2_	4.6-Dihydroxyquinoline	C05639	Tryptophan metabolism
M402T328	_nat._U	Unknown							
M132T32	Control	C_4_H_10_N_3_O_2_	[M+H]l+	3.2	17.7	C_4_H_9_N_3_O_2_	Creatine	C00300	Glycine, arginine, proline, serine metabolism
M143T32	Control	C_7_H_15_N_2_O	[M+H−H_2_O]l+	25.8	1.6	C_7_H_17_N_2_O_2_	N6-Methyl-l-lysine	C02728	
M185T351	_nat._U	Unknown							
M157T276	_nat._U	C_8_H_13_O_3_	[M+H−H_2_O]l+	26.8	0.6	C_8_H_14_O_4_	Suberic acid aglycone	C08278	DIcarboxylic acids
M209T66	Control	C_10_H_13_N_2_O_3_	[M+H−H_2_O]l+	8.8	11.3	C_10_H_14_N_2_O_4_	Porphobilinogen	C00931	Prophyrin metabolism
M162T107	Control	C_9_H_8_NO_2_	[M+H]l+	5.9	5.1	C_9_H_6_NO_2_	4.6-Dihydroxy quinoline	C05639	Tryptophan metabolism
M129T328	_nat._U	C_7_H_13_O_2_	[M−HCO_2_H+H]1+	8.3	6.3	C_8_H_14_O_4_	Suberic acid moiety(desulfated)	C08278	Dicarboxylic acids
M175T277	_nat._U	C_8_H_15_O_4_	[M+H]l+	18.6	3.9	C_8_H_14_O_4_	Suberic acid	C08278	Dicarboxylic acids
M175T451	Control	C_13_H_19_	[M−2H_2_O+H]1+	10.4	1.6	C_13_H_22_O_2_	3Z.5E-tridecadienoic acid aglycone (glucuronide)		Lipid metabolism
M257T384	Control	Unknown aglycone							
M299T496	Control	C_18_H_19_O_4_	[M+H]l+	5.6	2.3	C_18_H_18_O_4_	7C-aglycone	HMDB04808	Phylloquinone (Vitamin K1) and menaquinones (Vitamin K2) metabolism
M164T347	_nat._U	Unknown							
M164T420	Control	C_9_H_10_NO_2_	Desulfated moiety [M+H]1+	33.7	5.5	C_9_H_9_NO_2_	4-(3-Pyridyl)-3-butenoic acid moiety (desulfated)	HMDB01424	Nicotine degradation II and nicotine degradation III
M316T348	_nat._U	Unknown							
M342T284	Control	Unknown							
M161T287	_nat._U	C_8_H_15_O_2_	[M+H]l+	15.4	9.9	C_8_H_16_O_3_	Hydroxyoctanoic acid (aglycone)	HMDB00711	Fatty acid metabolism
M143T287	_nat._U	C_8_H_15_O_2_	[M+H−H_2_O]l+	15.4	9.9	C_8_H_16_O_3_	Hydroxyoctanoic acid (aglycone)	HMDB00711	Fatty acid metabolism
M175T269	_nat._U	C_8_H_15_O_4_	[M+H]l+	33.2	0.2	C_8_H_14_O_4_	Suberic acid moiety (cysteine-glycine conjugate)	C08278	
M389T275	_nat._U	Unknown							
M311T347	_nat._U	Unknown							
M181T346	_nat._U	Unknown							
M447T410	Control	C_15_H_11_O_5_	[M+H]l+	6.7	4.4	C_15_H_10_O_5_	Flavonoid-glucuronide	C10023	Flavonoids
M164T188	Control	C_9_H_10_O_2_	[M+H]l+	13.4	15.8	C_9_H_9_NO_2_	4-(3-Pyridyl)-3-butenoic acid	HMDB01424	Nicotine degradation II and nicotine degradation III
M461T347	Control	C_16_H_13_O_5_	[M+H]l+	10.3	11.7	C_16_H_12_O_5_	Flavonoid-glucuronide	C10047	Flavonoids
M153T47	_nat._U	C_7_H_9_N_2_O_2_	[M+H]l+	13.1	2.5	C_7_H_8_N_2_O_2_	Nl-Methyl-2-pyridone-5-carboxamide	C05842	Nicotinate and nicotinamide metabolism, deterioration of kidney function
M206T158	Control	C_10_H_8_NO_4_	[M+H]l+	8.3	2.8	C_10_H_7_NO_4_	6-hydroxykynurenate	C05663	Tryptophan metabolism
M146T262	_nat._U	C_9_H_8_NO	[M−CO_2_+H]l+	4.2	4.1	C_10_H_9_NO_3_	5-Hydroxyindoleacetic acid	C05635	Tryptophan metabolism
M385T423	_nat._U	Unknown glucuronide							
M174T296	_nat._U	C_10_H_8_NO_2_	[M−H_2_O+JH]1+	10.5	2.2	C_10_H_7_NO_2_	Quinaldic acid	C06325	Tryptophan catabolism via kynurenic acid
M162T164	_nat._U	C_9_H_8_NO_2_	[M−CO_2_+H]l+	5.6	2.1	C_10_H_7_NO_4_	Xanthurenic acid	C02470	Tryptophan catabolism
M146T323	Control	Unknown							
M387T432	_nat._U	C_16_H_28_NaO_9_	[M+Na]l+	15.6	13.5	C_10_H_20_O_3_	Hydroxy-decanoic acid aglycone (glucuronide)	LMFA0105015	Fatty acid metabolism
M197T324	_nat._U	Unknown							
M373T270	_nat._U	C_17_H_21_N_4_O_6_	[M+H]l+	6	2.1	C_17_H_2_ON_4_O_6_	Riboflavin	C00255	Riboflavin metabolism

Listed from the PLS-DA model built on the variables exhibiting a VIP score ≥1.8

^a^Mostly abundant in indicated sample type

^b^Formula calculated from the accurate mass and the sigma-fit score by data analysis

^c^Mass accuracy related to isotopic decay (sigma fit < 20 is considered as relevant)

#### Urinary metabolites responsible for discriminating between control and contaminated rats

Of the top 40 discriminatory metabolites in urine samples (Table 2), 36 were tentatively annotated with the MZedDB database browser, and 28 had a corresponding KEGG ID. Fourteen of these 28 were mapped in pathways according to the KEGG Mapper search: 8 in the tryptophan metabolism pathway, 7 in global metabolic pathways, and 2 in the nicotinate and nicotinamide metabolism pathway. Eight of these 14 molecules were available from suppliers and purchased for further analytical confirmation.

N1-methylnicotinamide (also called trigonellinamide), creatine and 4,6-dihydroxyquinoline were proposed to be [M+H]1+ forms of the molecules, so the target masses for MS/MS analyses were the same for the standards and the U50 urine sample, which had the highest intensities for these metabolites.

The Metlin spectrum search result for *N*-methylnicotinamide was a match for both the standard and U50 sample (supplemental Fig. 1) samples. In U50, the main fragments obtained at 20 eV collision energy were the precursor (*m*/*z* 137.073) and *m*/*z* 94 (loss of the carbamaldehyde group). Such a spectrum was also found in MassBank and HMDB. Matched retention time at 30 s between the authentic standard and the *m*/*z* 137.07 ion trace in the urine samples was additionally confirmatory.

The Metlin spectrum search result for creatine was also a match for both the standard and U50 (supplemental Fig. 2) samples. The main fragments obtained at 20 eV collision energy were the precursor (*m*/*z* 132.07) and *m*/*z* 90.053 (loss of the methanimidamide group). Such a spectrum was also found in MassBank and HMDB. Another fragment, *m*/*z* 111.057, is present in U50 at a high intensity (and also in MassBank database at a much lower intensity). The commensurate increase of creatine peak height at 31 s by spiking the urine sample with various amount of an authentic standard also brought a supplementary chromatographic confirmation.

There was no spectrum match for 4,6-dihydroxyquinoline in either Metlin, MassBank or HMDB, due to the lack of MS/MS data in these databases for this particular compound. Nevertheless two quite superimposable fragment spectra were obtained for the standard and U50 samples at 138.6 s retention time (supplemental Fig. 3). The main fragments obtained at 20 eV collision energy were the precursor ion (*m*/*z* 162.05) and a fragment at *m*/*z* 144.05 (loss of a water molecule). Spiking the urine sample with the authentic standard also brought a chromatographic confirmation.

MZedDB proposed adduct/fragment ions for 5-hydroxyindoleacetic acid, the other tentatively annotated molecules. The Metlin spectrum search result was a match for both the standard and (supplemental Fig. 4) samples: both fragment and precursor were clearly co-detected in the urine sample, at the same retention time. The main fragments obtained at 10 eV collision energy were the precursor (*m*/*z* 192.07) and an ion at *m*/*z* 146.06 (loss of a formic acid molecule). Such a spectrum was also found in HMDB, but there was no QTOF spectrum in MassBank for this metabolite. At 30 eV, 2 other fragments appeared in the standard: *m*/*z* 128.05 (loss of a carbonic acid) and 118.07 (loss of a formaldehyde group from the 146.06 ion). Again spiking the urine sample with increasing amount of an authentic standard additionally argued for identification (supplemental Fig. 4).

According to the minimum metadata relative to metabolite identification of non-novel compounds proposed by the metabolomics standard initiative (Sumner et al. 2007), we have thus far identified 4 metabolites, the concentrations of which were modified after chronic low-dose uranium contamination: 1-methylnicotinamide, creatine, 4,6-dihydroxyquinoline (reduced concentration in the urine of contaminated rats) and 5-hydroxyindoleacetic acid (increased concentration in the urine of the contaminated rats).

## Discussion

Thus far, experimental studies of rat models exposed to chronic low-dose uranium contamination have shown metabolic changes without the onset of any morbidity. For example, targeted analysis of biochemical systems has revealed deregulation of the xenobiotic, cytochrome P450, Vitamin D, cholesterol, iron, and acetyl choline metabolisms (Berradi et al. 2008; Bussy et al. 2006; Gueguen et al. 2007; Racine et al. 2010; Souidi et al. 2005; Tissandie et al. 2007). Nevertheless, documentation of the effects of low-dose _nat._U contamination remains poor, likely due to the difficulty in demonstrating any health impact of a compound absorbed at a very low dose, possibly over a long period. A very sensitive method is required to investigate such potentially low-grade effects. We previously applied untargeted metabolomics in similar conditions to examine chronic low-dose ^137^Cs contamination and found that this approach showed promise for revealing metabolic disruption where other conventional methods failed to discriminate between exposed and unexposed rats (Grison et al. 2012).

Here, we sought to evaluate the effects of chronic low-dose exposure to _nat._U on rat health, both with conventional toxicological tests, such as organ weighing and urine/plasma biochemical assays, and with a metabolomic approach based on analysis of urinary LC–MS profiles. To mimic the potential natural exposure of human populations, rats were chronically contaminated through drinking water at a daily intake of about 1 mg per animal. This dose corresponds to three times as much as the maximum natural concentration measured in Finnish wells and can be considered a realistic level of natural contamination (Salonen 1994). Moreover, no renal toxicity has been demonstrated at this dose in rats.

As previously described after 9 months of uranium contamination (Souidi et al. 2005), our results confirmed the absence of disease in these animals. Measurements of standard clinical biomarkers and biometric variables during and after treatment were similar in both experimental groups. We did not observe any problem such as anorexia, substantial weight loss, or macroscopically observable tissue damage (including in the kidneys, which are well known as a biological target of uranium in acute high-dose contamination) and were thus able to rule out any deterioration in health status. Nor did we observe any imbalance between the groups for CBC, in hepatic, heart, and kidney enzyme activities, or in lipid, protein, and ion concentrations in plasma. Markers of potential nephropathy, such as urine volume, liver enzyme concentration, carbohydrates and creatinine clearance were also measured and showed no changes (the few statistically significant changes observed nonetheless still remained within the range of physiologically normal).

It is well recognized that uranium has a dual toxicity, radiological (as an alpha emitter) and chemical (as a heavy metal) (Craft et al. 2004). When it includes substantial proportions of isotope 234 or 235, as enriched uranium does, it may be more radiologically than chemically toxic. For depleted or _nat._U, however, chemical toxicity is more important. Our data confirmed the absence of both types of toxicity. In fact, the low renal burden of uranium (around 300 ng/g of tissue) in this study, compared to the 3 μg/g value traditionally assumed as the threshold of renal toxicity (Leggett 1989), confirms the low-dose nature of the contamination of the rats in this study, as does the very low total absorbed dose calculated (150 μGy). In conclusion, this level of contamination is consistent with the measured renal effects, which did not demonstrate any associated disease or deterioration in general health status after 9 months of contamination with _nat._U.

Nonetheless, the absence of toxicity does not imply a lack of biological effects that might be detected by more sensitive techniques. This was clearly demonstrated when we used metabolomics to identify chronic low-dose exposure to cesium 137. As with ^137^Cs, the metabolomics approach applied here was quite able to discriminate the control animals from the uranium-contaminated rats. Uranium, as a heavy metal, accumulates specifically in target organs, especially the kidneys (Paquet et al. 2006). The significant discrimination between _nat._U non-exposed and exposed rats that we found in urine thus appears consistent with tubular accumulation of uranium and its consequent nephrotoxicity on exposure to higher doses (Blantz 1975; Haley et al. 1982). A similar metabolomics plasma investigation would be nevertheless worth to perform.

The most discriminatory metabolite was identified as N1-methylnicotinamide, found at a concentration seven times higher in the urine of control versus contaminated rats. A decrease in this metabolite has previously been described as an indicator of induced nephropathy, by administration of a single high dose of uranium (Shim et al. 1984). Our results might thus suggest early biological nephron response after 9 months of chronic low-dose ingestion of _nat._U. They also indicate that the nicotinamide pathway is a potential target for uranium. For instance, the urine content of several metabolites belonging to this pathway were changed by _nat._U contamination. Among them was N1-methyl-pyridone-5carboxamide that increased by _nat._U (Table 2), which is the product of N1-methylnicotinamide oxydo-reduction by the aldehyde oxidase EC:1.2.3.1 (supplemental Fig. 5). Thus suggesting that this converting enzyme can be a target for _nat._U biological effect. In addition, as pointed out above uranium interferes with the cytochrome P450, Vitamin D, cholesterol, and xenobiotic metabolisms: all these pathways use the enzyme cofactor nicotinamide adenine dinucleotide phosphate (NADP). Further targeted searches on this pathway would probably be especially useful to decrypt the underlying mechanisms by which uranium acts on these metabolisms.

**Fig. 3:**
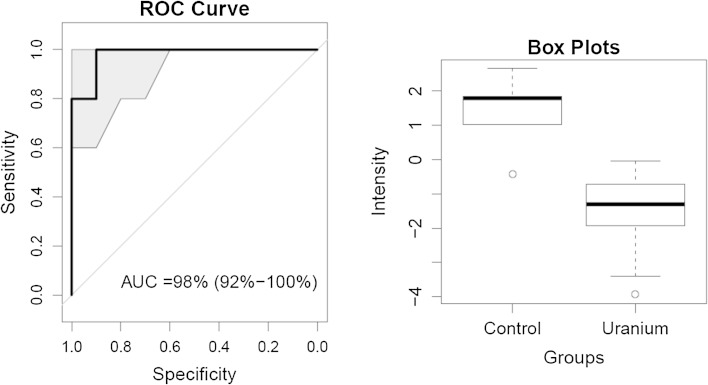
Receiver operating characteristic (ROC) curve, area under the curve (AUC), and *box plots* of the composite score built on the top 40 metabolites discriminating the control from the contaminated rats

We also identified creatine, 4,6-dihydroxyquinoline, and 5-hydroxyindoleacetic acid as highly discriminatory features (ranked 6th, 11th, and 32nd in the PLS-DA model, respectively). Based on these identified metabolites and 10 tentatively annotated ones (based on database searching), chronic low-dose _nat._U contamination appears to affect mainly the tryptophan and the nicotinamide metabolism pathways. N1-methylnicotinamide alone allowed 100 % discrimination with 100 % specificity and selectivity. Nevertheless, the risk of inaccuracy in relying on only one bioindicator could be high, since conditions other than uranium contamination might affect the level of this metabolite in urine. It would thus be wiser to use a combination of bioindicators, so that the potential failure of one would be offset by the others in the calculation of a global score reflecting uranium contamination more specifically. Such a strategy would be advantageous for designing a diagnostic test. In that perspective, we used the top 40 discriminatory metabolites from the PLS-DA model to build a composite score, and calculated its AUC under the ROC curve; this reached 98 % (CI 92–100 %) (Fig. 3).

In conclusion, this study confirms that the application of metabolomics is relevant to the field of chronic low-dose radiotoxicology, more so than more traditional techniques that lack sensitivity and seem unsuitable to exploring relatively minor or uneventful biological effects. Further work is needed to validate our findings. Such work should include a replication study with validation cohorts, radionuclide dose–response assessment, contamination kinetics, and a comparison of response by sex to investigate the physiological mechanisms responsible for the biological changes highlighted by metabolomics. The intensity of the biological effects measured and whether it exceeds the threshold of disease must also be clarified.

The noninvasive use of urine as a reproducible biofluid and validation of our other findings should make it possible to design a diagnostic tool able to sort populations potentially contaminated by environmental _nat._U.

## Electronic supplementary material

Below is the link to the electronic supplementary material.

Supplementary material 1 (DOC 729 kb)
