# Supplementary material for: Metabolomics identifies a biological response to chronic low-dose natural uranium contamination in urine samples
Source: Metabolomics. 2013 May 21;9(6):1168–80. doi: 10.1007/s11306-013-0544-7 (PMC3825637; doi:10.1007/s11306-013-0544-7)

**Supplemental Table 1.** Mean ± SEM of complete blood count in control and contaminated (_nat._U) groups at 9 months. Number of rats for each measurement is indicated in parentheses.

**Supplemental Figure 1.** urine sample spiked with an authentic standard of 1-methylnicotinamide and MS/MS spectra comparison between the standard and the isolated m/z 137.07 ion at 20eV CID energy.

**Supplemental Figure 2.** urine sample spiked with increasing amount of an authentic standard of creatine and MS/MS spectra comparison between the standard and the isolated m/z 192.068 ion at 20eV CID energy.

**Supplemental Figure 3.** urine sample spiked with increasing amount of an authentic standard of dihydroxyquinoline and MS/MS spectra comparison between the standard and the isolated m/z 162.057 ion at 20eV CID energy.

**Supplemental Figure 4.** urine sample spiked with increasing amount of an authentic standard of 5-hydroxyindole acetic acid and MS/MS spectra comparison between the standard and the isolated m/z 162.057 ion at 10eV CID energy.

**Supplemental Figure 5.** Nicotinate and nicotamide metabolism


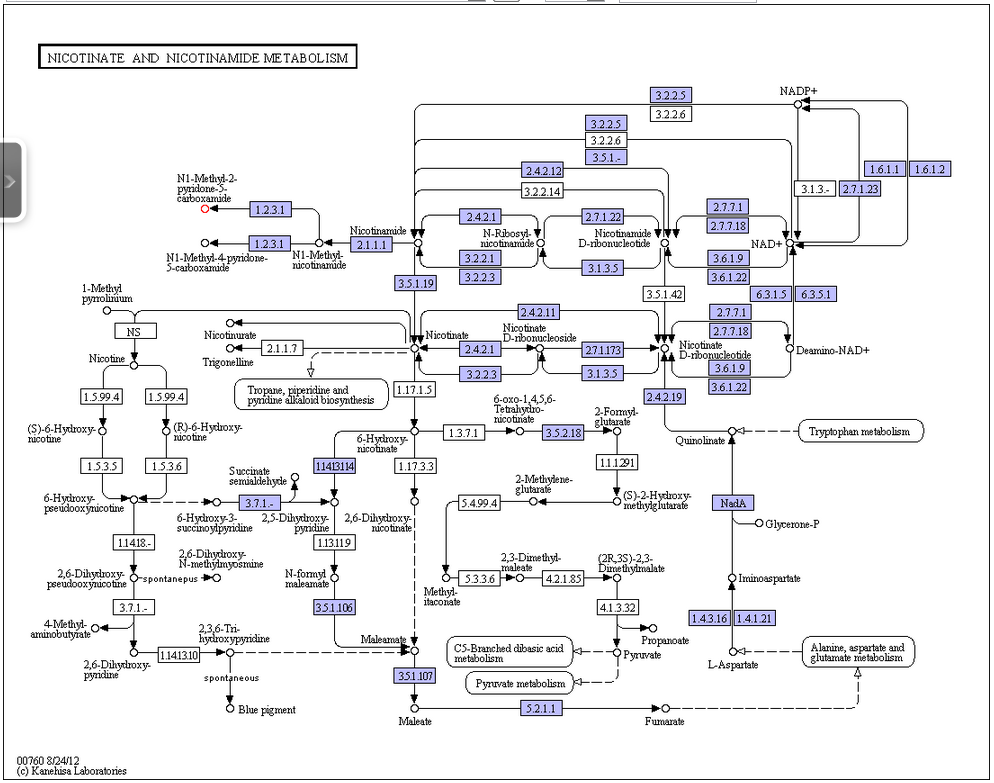

Supplement: Supplementary file 1 — Supplementary material 1 (DOC 729 kb) [file 11306_2013_544_MOESM1_ESM.doc]
